# Dependency relationships within the fission yeast polarity network

**DOI:** 10.1002/1873-3468.13180

**Published:** 2018-07-13

**Authors:** Matthew Johnson, Daniel P. Mulvihill

**Affiliations:** ^1^ School of Biosciences University of Kent Canterbury UK

**Keywords:** actin, fission yeast, microtubules, polarised growth, *Schizosaccharomyces pombe*

## Abstract

The ability to regulate polarised cell growth is crucial to maintain the viability of cells. Growth is modulated to facilitate essential cell functions and respond to the external environment. Failure to do so can lead to numerous developmental and disease states, including cancer. We have undertaken a detailed analysis of the regulatory interplay between molecules involved in the regulation and maintenance of polarised cell growth within fission yeast. Internally controlled live cell imaging was used to examine interactions between 10 key polarity proteins. Analysis reveals interplay between the microtubule and actin cytoskeletons, as well as multiple novel dependency pathways and feedback networks between groups of proteins. This study provides important insights into the conserved regulation of polarised cell growth within eukaryotes.

## Abbreviations

**EMM2**, Edinburgh minimal media

**NETO**, New End Take Off

Cell polarity is a fundamental property for all living cells, as control of cell shape is crucial for cell survival. Polar growth determines shape generation in a variety of cell types including neurons, epithelial cells and yeast 1, 2, 3. This spatially coordinated growth pattern is critical for the function of cells, and within a multi‐cellular context is crucial for the proper development of the metazoan organism. At the molecular level, polarised cell growth is determined by the spatial arrangement of key proteins which form functionally specialised complexes within the cytosol and at the cell membrane. The sub cellular localisation of these polarity determinants facilitates a diverse variety of cellular processes such as differentiation, membrane growth, cell migration, neuronal development, activation of the immune response and transport of vesicles across layers of cells. To acquire polarity, cells must break symmetry, which is often achieved through cooperation between the actin and microtubule cytoskeletons. This brings about an asymmetric distribution of organelles and polarity factors within the cell to promote the establishment of a polarised pattern of cell growth.

*Schizosaccharomyces pombe* is an excellent model system in which to study the establishment and maintenance of cell polarity and shape. Polarised growth in this unicellular yeast is similar to that of neuronal cells, in that they grow exclusively from their cell tips. This mono‐axial growth pattern is regulated in a cell cycle dependent manner. Upon cell division, the fission yeast cells grow exclusively from the ‘old’ end of the cell that originally formed one end of the parental cell. Then at a critical point during G2, in a process referred to as New End Take Off (NETO), cell growth becomes bipolar until the onset of mitosis 4. This switch from monopolar to bipolar growth correlates precisely with a parallel re‐distribution of actin 5. Although actin is essential for polar growth it is not sufficient to establish a bipolar growth pattern, which requires the activity of the microtubule cytoskeleton, which also facilitates the distribution of polarity determinants to the cell tips and thereby promotes actin assembly at these sites of cell growth.

These polarity determinants are made up from a wide variety of families of proteins conserved from fission yeast to humans, which play diverse roles in maintaining polarised cell growth. This conservation, combined with the clear phenotypic outcomes observed in cells lacking polarity factors (develop abnormal cell shapes becoming bent, T‐shaped, spherical, etc.) 6 and ease with which it allows itself to live cell imaging has made *S. pombe* an excellent model system in which to study the molecular regulation of cell growth and division. The cellular organisation of many of these key polarity molecules have been characterised, however, the impact each molecule has upon the distribution of each of the other polarity proteins is currently unknown.

Fission yeast microtubules act as the primary vehicle upon which key polarity determinant proteins are delivered to the cell tip. These polymers are stabilised by Mal3 and Tip1, homologues of human EB1 and CLIP170 proteins 7, 8, 9, which are delivered to microtubule +ends by the Kip2 related kinesin, Tea2 10, 11. Here they complex with Tea1, a landmark Kelch repeat containing protein, and with Tea2 and Tip1 travel on the ends of microtubules to the end of the cell, where they are deposited at the cell cortex 12. Here these three molecules interact with further proteins, including Bud6, Tea3, Tea4 and Mod5 13, 14, 15, 16, 17, to promote a tightly defined polar region of growth and together play a key role in initiating the transition from monopolar to bipolar growth. While polar recruitment of the actin nucleating formin, For3, is dependent upon Cdc42 18, Tea1 and Tea4 regulate its switch to bipolar distribution at NETO 13. These For3 nucleated actin polymers provide a track on which the class V myosin Myo52 can travel and deliver cargoes (e.g. vesicles) to facilitate the synthesis of the new cell tip 19, 20. These interactions have been established in diverse labs using a variety of techniques (e.g. co‐immunoprecipitation, pull down and 2‐hybrid assays). Critically individual localisation dependencies determined on cells subjected to diverse growth conditions, imaging technologies and techniques, making it challenging to define a global picture of localisation dependencies between each key polarity protein with any significant level of confidence.

We have undertaken a detailed analysis of the interactions and regulatory interplay between ten key molecules involved in the regulation and maintenance of polarised cell growth. Through the systematic three‐dimensional localisation of 10 polarity determinants, a detailed interdependence network has been characterised. This reveals a series of interconnecting positive and negative feedback loops and pathways that coalesce to provide a robust and precise mechanism for modulating cytoskeleton organisation and providing a framework for regulating polarised cell growth.

## Materials and methods

### Strains and cell cultures

All strains used in this study are listed in Table S1. Cells were cultured at 25°C in Edinburgh minimal media (EMM2) supplemented with appropriate amino acids 21. Strains in which the *tea2* allele was entirely replaced with the hygromycin resistance gene (*hphMX6*) were created as described previously 22. All cells were cultured exponentially for 48 hr before microscopy analysis.

### Microscopy and image analysis

Live cell imaging was undertaken as described previously 23 with cells mounted directly from the culture (without centrifugation) onto lectin‐coated coverslips and into a Bioptechs FCS2 chamber (Bioptechs, Butler, PA). The intensity of GFP signal was measured with metamorph software (Molecular Devices, Sunnyvale, USA) from maximum projections generated from 31 × 0.2 μm separated Z slice images. The average and maximum signal intensities were measured within 3 μm diameter circular regions of interest within the image background, both ends (‘End1’ assigned to cell end with brighter GFP signal) and within non‐foci containing regions of the cytoplasm of each cell. The cell measurements were subsequently background corrected. The GFP signal for a specific protein was determined from more than 100 cells of each deletion strain and compared with > 100 wild‐type cells acquired from the same coverslips. These values were used to calculate average differences in relative average FP signal at the cell tips and cytosol between wild‐type and deletion cells. The raw image files acquired during this study are stored at the Kent Data Repository and are available online at https://data.kent.ac.uk/45/.

## Results and Discussion

**Figure 1:**
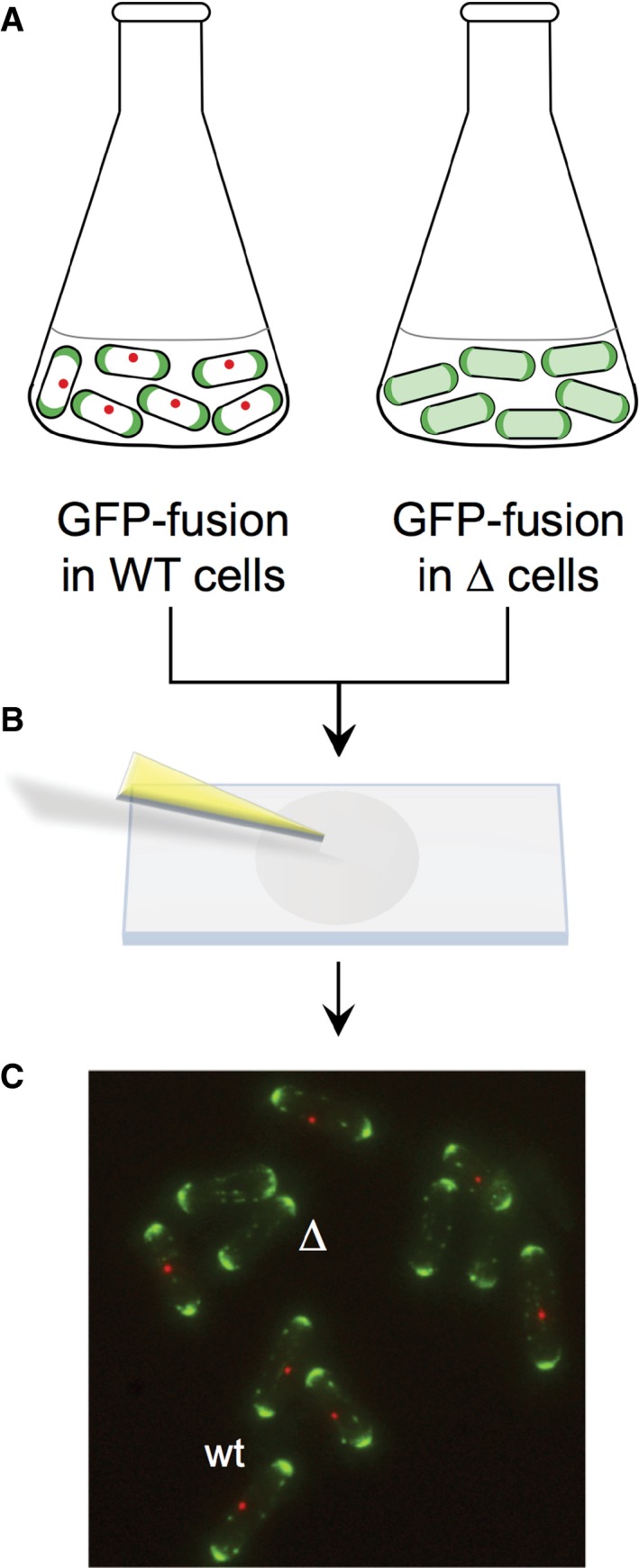
Strategy for generating control containing images for consistent signal intensity analysis. (A) Cultures of fission yeast cells expressing a GFP protein of interest in either a wild‐type (co‐expressing an unrelated red FP labelled protein) or a specific gene deletion background are grown at mid‐log phase for 48 hr, and (B) mixed and mounted onto lectin‐coated coverslips. (C) Multi‐z‐slice image datasets of individuals group of cells were captured and used to simultaneously generate maximum projections of the 3d data from wild‐type and deletion cells.

We wished to establish how each of the key fission yeast polarity proteins affect the recruitment of each of the other proteins within the polarity network within a single study in order to gain an understanding of the regulation of this complex molecular signalling system. In order to facilitate this, we established a live cell imaging‐based assay where relative differences in localisation signal intensity could be directly compared between different strains with a high degree of confidence and reproducibility. Strains were used in which GFP cDNA was fused to the gene encoding for a specific polarity marker at the genomic locus and were thus subject to endogenous transcriptional control. Each of these GFP labelled alleles were crossed with strains in which genes encoding for each of the other polarity proteins had been deleted. At the same time a series of wild‐type comparison strains were generated, each expressing the essential spindle pole body component Sid4 24 fused to tdTomato in combination with the GFP labelled polarity protein being analysed. During subsequent live cell imaging experiments, the fluorescence signal from the GFP labelled polarity marker was simultaneously examined in both wild‐type cells, co‐expressing Sid4.tdTomato, and in cells in which a gene encoding for a different polarity marker had been deleted (Fig. 1). During image acquisition, each captured field of view contained a mixture of wild‐type and deletion cells, providing an internal control to allow a direct comparison between the GFP signal intensity from wild‐type and deletion strains. This provides confidence that any observed differences in signal between the observed strains are a consequence of the gene deletion and not due to variations in experimental conditions such as media, temperature, coverslip surface, fluctuations in light source intensity, or variation in settings of the imaging system. This provides a high degree of confidence and sensitivity in establishing statistically significant differences in signal between strains.

**Figure 2:**
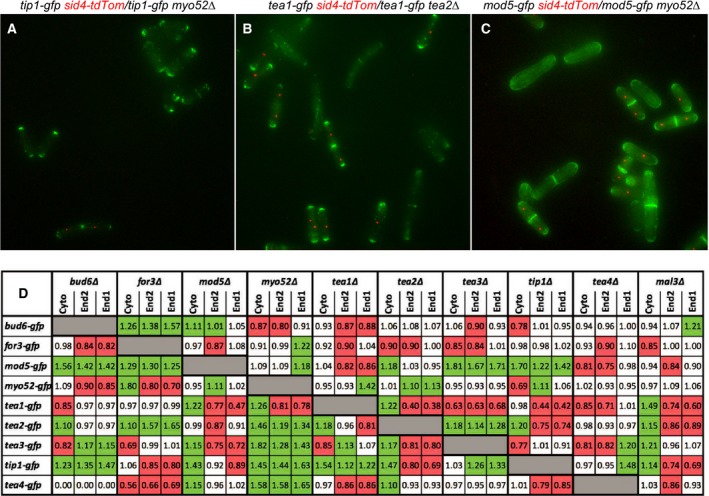
Polarity marker localisation dependency analysis. Maximum projection images showing localisation of Tip1‐GFP (A), Tea1‐GFP (B) and Mod5‐GFP (C) (green) captured simultaneously in cells with *myo52* ^*+*^ *and myo52∆ sid4.tdTomato* (red*)* (A and C) or *tea2* ^*+*^ *and tea2∆ sid4.tdTomato* (red*)* (B) genetic backgrounds. (C) Average differences (relative to wild‐type) in the GFP signal of different polarity marker proteins (rows) within the cytoplasm or each ends of cells lacking each of the other polarity proteins, or EB1 homologue (*mal3∆*) (columns). The matrix highlights significant increases (green) or decrease (red) in average relative signal between the deletion and wild‐type strains.

Images were acquired that allowed analysis of the distribution of each polarity protein in at least 100 cells from each deletion strain, and a comparable number of simultaneously imaged equivalent number of control wild‐type cells on the same coverslips (i.e. > 18 000 cells analysed in the course of this study). The raw image data (made available at https://data.kent.ac.uk/45/) were used to generate maximum projections from individual 31‐z slices and present 3d data as a single plane to allow analysis of total GFP signal within the cytoplasm of each cell analysed (e.g. Fig. 2A–C). This method was applied to systematically examine the cellular distribution of 9 separate polarity proteins and how they were affected by deleting genes encoding for each of 10 other polarity affecting proteins. Typical examples of maximum projections of each deletion and control strain mix are shown in the supplemental data (Figs S1–S9). From these data, the background corrected average fluorescence signal at the poles and medial cytosol were determined for more than 100 interphase cells for each deletion strain. These values were normalised to corresponding background corrected values obtained from equivalent wild‐type cells from the same images. Data for each GFP fusion and gene deletion combination were then combined to generate a detailed matrix providing quantification of how of each deletion impacted the monopolar and bipolar recruitment and cytoplasmic abundance of each of the polarity regulating proteins (Fig. 2D).

From these data we were able to identify negative and positive dependency relationships between each polarity protein. A negative dependency is illustrated by the effect of Myo52 on Tip1 recruitment to the cell tip (Fig. 2A). Significantly more Tip1 is observed at the poles of cells lacking Myo52, consistent with the role of this myosin V in preventing the build‐up of Tip1 through the regulated proteolysis of polar Tip1 25. Conversely an example of a positive dependency is provided by the reliance of Tea1 upon the kinesin microtubule motor, Tea2, for it to localise to the cell poles (Fig. 2B).

The data not only confirmed previous observations but provide evidence of as yet unrevealed relationships and regulatory mechanisms. For example this study is not only consistent with previous studies demonstrating a dependency of Tea1 upon Tea2, Tea3, Tea4, Tip1 and Mod5 10, 13, 14, 16, 17, 26, and a previously unobserved dependency upon Myo52 (Fig. 2D). Unsurprisingly, each of the microtubule targeted polarity markers failed to recruit to the cell pole in strains lacking the microtubule stabilising proteins Mal3 or Tip1, in which microtubules have reduced stability and are unlikely to grow long enough to contact the cell pole and deposit markers there (Fig. 2D) 7, 26. Similarly, the data are in agreement with studies that illustrate Mod5 is required to anchor the majority of the microtubule dependent polarity proteins to the polar plasma membrane 16, 17 However, the data reveal its own localisation is impacted by Bud6, Tea3 and Tip1, indicating an as yet unexplored regulatory complexes.

**Figure 3:**
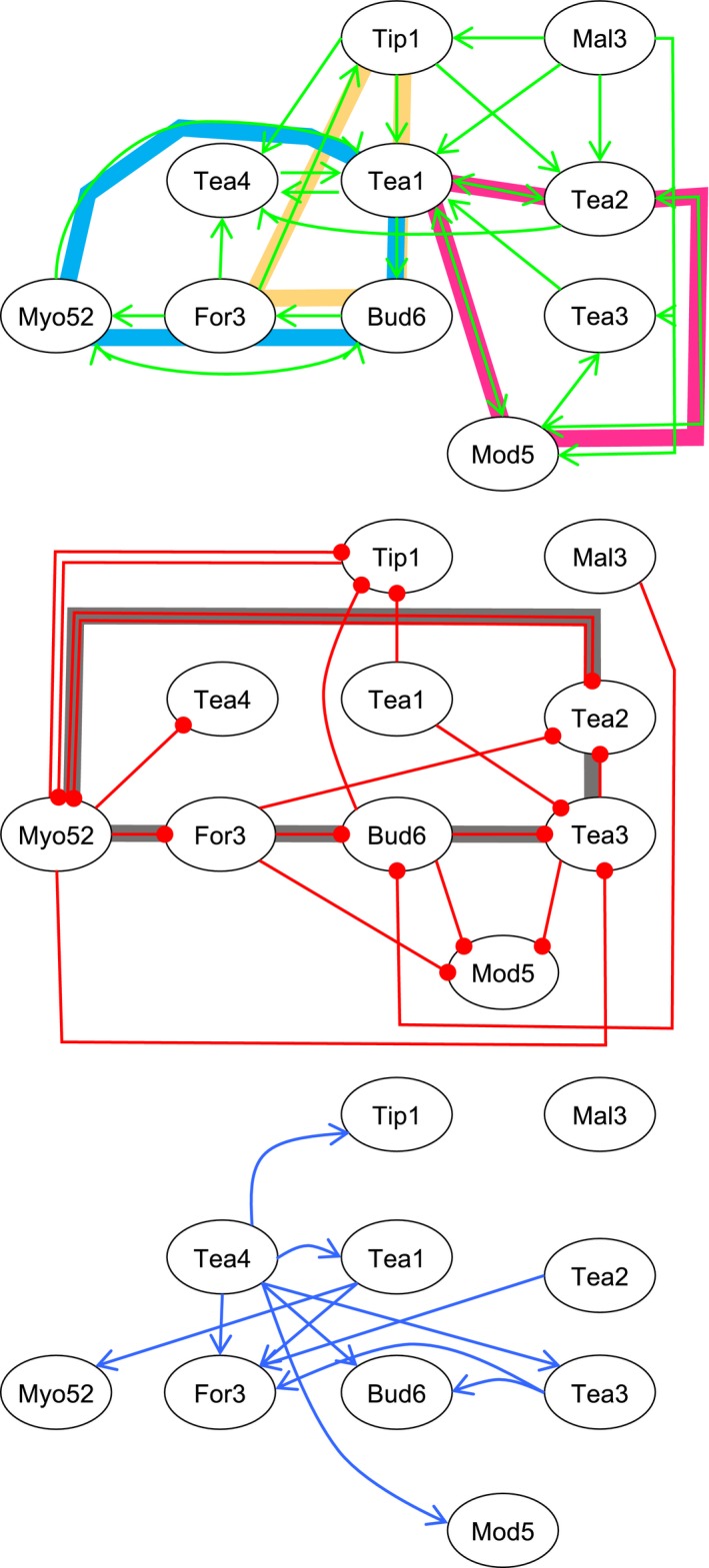
Recruitment dependency network of polarity proteins. Dependency networks determined from differences in average relative differences in polar localisations. Positive regulations (green arrows) require the upstream protein for polar localisation, whereas negative regulations (red arrows) require the upstream protein to reduce polar signal. Blue arrows signify dependence on upstream protein for switch from monopolar to bipolar distribution at NETO. Broad coloured lines highlight larger positive (cyan, yellow and magenta) and negative (grey) feedback network loops between proteins.

These examples provide validation of the strategy used as they are consistent with and also extend previous findings. The robust and consistent sensitive quantification of relative signals, allowed by this normalised averaging technique, reveals previously unobserved localisation dependencies between proteins, which are illustrated in the polarity marker recruitment dependency network (Fig. 3) generated from analysis complete dataset. Analysis of the network reveals different classes of feedback loops:

- Direct unidirectional feedback loop between two proteins, where protein A promotes the recruitment of protein B, which in turn prevents excess accumulation of A. Examples include interactions between Bud6 and For3, Tip1 and Tea1, For3 and Myo52, Tea3 and Tea1 or Mod5 and Tea3.
- Positive feedback amplification loop between two proteins, where proteins A and B promote the recruitment of each other. Examples include interactions between Tea1 and Tea4, Tea1 and Tea2, Tea1 and Mod5, or Bud6 and Myo52.
- Negative feedback reduction loop between two proteins, where proteins A & B inhibit accumulation of the other. Examples include interactions between Myo52 and Tea2.
- Larger positive and negative feedback loops between three or more proteins. The three positive loops (i. Tea1‐Bud6‐For3‐Myo52‐Tea1; ii. Tea1‐Bud6‐For3‐Tip1‐Tea1; iii. Tea1‐Tea2‐Mod5‐Tea1) each contains Tea1, making it core in the polarity networks. These and the negative feedback loop (Myo52‐For3‐Bud6‐Tea3‐Tea2‐Myo52) each provide mechanisms for bi‐directional crosstalk between the actin and microtubule cytoskeletons to allow modulation of each of their activities during polarised cell growth.

The network reveals some apparent contradictions. For example the localisation and movement of the Myo52 myosin is dependent upon For3‐nucleated actin filaments, the simple prediction would be that it would have the same effect as For3 on a subset of downstream proteins. However, this is not the case, potentially due to Myo52 preventing the build‐up of For3 at the cell tip 13. At the same time Myo52 prevents the build‐up of Tip1 (though facilitating its proteolysis), which is in turn part needed to deliver Tea1, another For3 effector, to the cell pole.

The data suggest that regulating the proteolysis of polarity determinants could provide a common mechanism for ensuring rapid turnover of proteins at the cell tips. This will allow the cell to maintain or modulate polarised cell growth in response to cell cycle progression and changes in the inter and extra cellular environment. In the absence of a protein that promotes proteolysis of a fluorescently labelled protein, a global increase in fluorescence signal (both cytoplasmic and polar) would be observed. Not only was Myo52 seen to be required to stabilise Tip1 levels in wild‐type cells 25, these data showed similar effects of Myo52 on the microtubule associated polarity proteins Tea2, Tea3, Tea4 (Fig. 2D), whereas For3 competes with and regulates the proteolysis and localisation of Bud6, Mod5 and Tea2. Conversely, the data suggest interactions between polarity proteins can also promote their stability. For example not only does Tea3 stabilise global Tea1 levels (Fig. 2D), but both For3 and Tea1 stabilise Tea4 levels. This is consistent with studies from the Chang lab 13 that indicate Tea4 facilitates formation of the Tea1‐Tea4‐For3 complex at the cell pole. These data presented here may indicate this complex is required to prevent proteolysis of Tea4, and may provide an insight into a dynamic system that allows a cell to rapidly switch from monopolar to bipolar growth pattern.

Interestingly Tea4, and to a lesser extent Tea3, drive the post NETO bipolar redistribution of proteins, directly, and sometimes indirectly which is consistent with previous observations 13, 25. We observed a significant difference in relative polar distribution of some proteins between the two ends within *tea1∆* (For3, Myo52, Tea2) cells, presumably via Tea4. While there was an overall reduction in Myo52 at both poles in *tea4∆* cells, and Bud6 in *tea1∆* cells, our analysis revealed no significant difference in monopolar vs. bipolar distribution of Bud6 in *tea4∆* cells. Surprisingly both Tip1 and Mal3 are required for microtubules to reach the end of the fission yeast cell, and both are required for the polar recruitment of Tea1, Tea2 and Tea4, proteins critical for regulating the switch between monopolar and bipolar growth. However, cells lacking Mal3 and Tip1 did not display significant differences in monopolar : bipolar distribution of the other polarity proteins studied here. This indicates NETO and polarised cell growth is not simply determined by delivery of proteins to the cell tip, but the cell length, interactions and regulatory signals they affect and but also signals affecting these polarity complexes at the cell end.

It is important to considerer localisation may not necessarily reflect only cellular function of the protein, as in some cases proteins may be able to undertake function without having observable discrete localisation. In addition, not all of the GFP labelled proteins are fully functional, as demonstrated by the synthetic phenotypes displayed by the *bud6‐gfp* and *mod5‐gfp* alleles in a variety of deletion strains (Figs 2C, S1 and S3).

In summary, we present a sensitive system‐based approach for establishing a detailed localisation dependency network between a large array of proteins. This methodology can be applied to the study of the organisation of other networks in a variety of different organisms, although the molecular plasticity and experimental tractability of the yeast still make them the most attractive model system for large scale system based genetic approaches. With the development of automated image capture and image analysis techniques 27 for yeast, it is now possible to automate the work flow pipeline, allowing rapid acquisition and analysis of massive datasets, and provides the exciting prospect of establishing a global localisation dependency for the entire proteome.

## Author contributions

Conceptualization: DPM; Investigation: MJ; Data analysis: MJ and DPM; & DPM; Model development: DPM; Data curation: DPM; Manuscript preparation, reviewing and editing: DPM; Project administration: DPM; Funding acquisition: DPM.

## Supporting information


**Table S1.** Strains used in this study.

**Fig. S1.** Polarity marker localisation dependency of Bud6‐GFP.
**Fig. S2.** Polarity marker localisation dependency of For3‐GFP.
**Fig. S3.** Polarity marker localisation dependency of Mod5‐GFP.
**Fig. S4.** Polarity marker localisation dependency of Myo52‐GFP.
**Fig. S5.** Polarity marker localisation dependency of Tea1‐GFP.
**Fig. S6.** Polarity marker localisation dependency of Tea2‐GFP.
**Fig. S7.** Polarity marker localisation dependency of Tea3‐GFP.
**Fig. S8.** Polarity marker localisation dependency of Tip1‐3GFP.
**Fig. S9.** Polarity marker localisation dependency of Tea4(Wsh3)‐GFP.
