# Supplementary material for: Dependency relationships within the fission yeast polarity network
Source: FEBS Lett. 2018 Jul 13;592(15):2543–9. doi: 10.1002/1873-3468.13180 (PMC6120479; doi:10.1002/1873-3468.13180)
Supplement: Supplementary file 1 — Table S1. Strains used in this study. [file FEB2-592-2543-s001.pdf]

Supplemental Table 1: *Strains used in this study*

Genotype	Source
ura4.d18 leu1.32 ade6.M210	Lab stock
ura4.d18 leu1.32 his2.d1	Lab stock
for3::kanMX6 ura4.d18 leu1.32 ade6.M210	[1]
bud6::kanMX6	[2]
myo52::URA4 ura4.d18 leu1.32 ade6.M210	[3]
mal3::URA4 ura4.d18 leu1.32 ade6.M210	[4]
tip1::kanMX6 leu1.32 ura4.d18	[4]
gef1::URA4 leu1.32 ura4.d18	[5]
mod5::kanMX6 leu1.32 ura4.d18 ade6.M216	[6]
tea1::URA4 ura4.d18	[7]
tea2::hphMX6 leu1.32 ura4.d18	This Study
tea3::kanMX6 leu1.32 ura4.d18	[8]
wsh3::natMX6 leu1.32 ura4.d18	[9]
for3.gfp:kanMX6 leu1.32 ura4.d18	[1]
bud6.gfp:kanMX6 leu1.32 ura4.d18	[2]
myo52.gfp:kanMX6 ade6.M210 leu1.32 ura4.d18	[3]
mal3.gfp:URA4 ura4. leu1.32 ura4.d18	[4]
tip1.4gfp:kanMX6 leu1.32 ura4.d18	[4]
nmt81mod5.gfp:kanMX6 ade6.M210 leu1.32 ura4.d18	[6]
tea1.gfp:URA4 leu1.32 ura4.d18	[7]
tea2.gfp:kanMX6 leu1.32 ura4.d18his3.d1	[10]
tea3.gfp:kanMX6 leu1.32 ura4.d18	[8]
wsh3.gfp:URA4 leu1.32 ura4.d18	[9]
for3::kanMX6 bud6.gfp:kanMX6 leu1.32 ura4.d18	This Study
for3::kanMX6 myo52.gfp:kanMX6 leu1.32 ura4.d18	This Study
for3::kanMX6 mal3.gfp:URA4 ura4.d18 leu1.32 ura4.d18	This Study
for3::kanMX6 tip1.4gfp:kanMX6 leu1.32 ura4.d18	This Study

for3::kanMX6 nmt81mod5.gfp:kanMX6 leu1.32 ura4.d18	This Study
for3::kanMX6 tea1.gfp:URA4 ura4.d18 leu1.32 ura4.d18	This Study
for3::kanMX6 tea2.gfp:kanMX6 leu1.32 ura4.d18	This Study
for3::kanMX6 tea3.gfp:kanMX6 leu1.32 ura4.d18	This Study
for3::kanMX6 wsh3.gfp:URA4 leu1.32 ura4.d18	This Study
bud6::kanMX6 for3.gfp:kanMX6 leu1.32 ura4.d18	This Study
bud6::kanMX6 myo52.gfp:kanMX6 leu1.32 ura4.d18	This Study
bud6::kanMX6 mal3.gfp:URA4 leu1.32 ura4.d18	This Study
bud6::kanMX6 tip1.4gfp:kanMX6 leu1.32 ura4.d18	This Study
bud6::kanMX6 nmt81mod5.gfp:kanMX6 leu1.32 ura4.d18	This Study
bud6::kanMX6 tea1.gfp:URA4 leu1.32 ura4.d18	This Study
bud6::kanMX6 tea2.gfp:kanMX6 leu1.32 ura4.d18	This Study
bud6::kanMX6 tea3.gfp:kanMX6 leu1.32 ura4.d18	This Study
bud6::kanMX6 wsh3.gfp:URA4 leu1.32 ura4.d18	This Study
myo52::URA4 for3.gfp:kanMX6 leu1.32 ura4.d18	Lab stock
myo52::URA4 bud6.gfp:kanMX6 leu1.32 ura4.d18	This Study
myo52::URA4 mal3.gfp:URA4 leu1.32 ura4.d18	Lab stock
myo52::URA4 tip1.4gfp:kanMX6 leu1.32 ura4.d18	Lab stock
myo52::URA4 nmt81mod5.gfp:kanMX6 leu1.32 ura4.d18	Lab stock
myo52::URA4 tea1.gfp:leu1.32 ura4.d18	Lab stock
myo52::URA4 tea2.gfp:kanMX6 leu1.32 ura4.d18	Lab stock
myo52::URA4 tea3.gfp:kanMX6 leu1.32 ura4.d18	This Study
myo52::URA4 wsh3.gfp:URA4 leu1.32 ura4.d18	This Study
mal3::URA4 for3.gfp:kanMX6 leu1.32 ura4.d18	This Study
mal3::URA4 bud6.gfp:kanMX6 leu1.32 ura4.d18	This Study
mal3::URA4 myo52.gfp:kanMX6 leu1.32 ura4.d18	Lab stock
mal3::URA4 tip1.4gfp:kanMX6 leu1.32 ura4.d18	This Study
mal3::URA4 nmt81mod5.gfp:kanMX6 leu1.32 ura4.d18	This Study
mal3::URA4 tea1.gfp:URA4 leu1.32 ura4.d18	This Study
mal3::URA4 tea2.gfp:kanMX6 leu1.32 ura4.d18	This Study

mal3::URA4 tea3.gfp:kanMX6 leu1.32 ura4.d18	This Study
mal3::URA4 wsh3.gfp:URA4 leu1.32 ura4.d18	This Study
tip1::kanMX6 for3.gfp:kanMX6 leu1.32 ura4.d18	This Study
tip1::kanMX6 bud6.gfp:kanMX6 leu1.32 ura4.d18	This Study
tip1::kanMX6 myo52.gfp:kanMX6 leu1.32 ura4.d18	Lab stock
tip1::kanMX6 mal3.gfp:URA4 leu1.32 ura4.d18	This Study
tip1::kanMX6 nmt81mod5.gfp:kanMX6 leu1.32 ura4.d18	This Study
tip1::kanMX6 tea1.gfp:URA4 leu1.32 ura4.d18	This Study
tip1::kanMX6 tea2.gfp:kanMX6 leu1.32 ura4.d18	This Study
tip1::kanMX6 tea3.gfp:kanMX6 leu1.32 ura4.d18	This Study
tip1::kanMX6 wsh3.gfp:URA4 leu1.32 ura4.d18	This Study
gef1::URA4 for3.gfp:kanMX6 leu1.32 ura4.d18	This Study
gef1::URA4 bud5.gfp:kanMX6 leu1.32 ura4.d18	This Study
gef1::URA4 myo52.gfp:kanMX6 leu1.32 ura4.d18	This Study
gef1::URA4 mal4.gfp:URA4 leu1.32 ura4.d18	This Study
gef1::URA4 tip1.4gfp:kanMX6 leu1.32 ura4.d18	This Study
gef1::URA4 nmt81mod5.gfp:kanMX6 leu1.32 ura4.d18	This Study
gef1::URA4 tea1.gfp:URA4 leu1.32 ura4.d18	This Study
gef1::URA4 tea2.gfp:kanMX6 leu1.32 ura4.d18	This Study
gef1::URA4 tea3.gfp:kanMX6 leu1.32 ura4.d18	This Study
gef1::URA4 wsh3.gfp:URA4 leu1.32 ura4.d18	This Study
mod5::kanMX6 for3.gfp:kanMX6 leu1.32 ura4.d18	This Study
mod5::kanMX6 bud6.gfp:kanMX6 leu1.32 ura4.d18	This Study
mod5::kanMX6 myo52.gfp:kanMX6 leu1.32 ura4.d18	This Study
mod5::kanMX6 mal3.gfp:URA4 leu1.32 ura4.d18	This Study
mod5::kanMX6 tip1.4gfp:kanMX6 leu1.32 ura4.d18	This Study
mod5::kanMX6 tea1.gfp:URA4 leu1.32 ura4.d18	This Study
mod5::kanMX6 tea2.gfp:kanMX6 leu1.32 ura4.d18	This Study
mod5::kanMX6 tea3.gfp:kanMX6 leu1.32 ura4.d18	This Study
mod5::kanMX6 wsh3.gfp:URA4 leu1.32 ura4.d18	This Study

tea1::URA4 for3.gfp:kanMX6 leu1.32 ura4.d18	This Study
tea1::URA4 bud6.gfp:kanMX6 leu1.32 ura4.d18	This Study
tea1::URA4 myo52.gfp:kanMX6 leu1.32 ura4.d18	This Study
tea1::URA4 mal3.gfp:URA4 leu1.32 ura4.d18	This Study
tea1::URA4 tip1.4gfp:kanMX6 leu1.32 ura4.d18	This Study
tea1::URA4 nmt81mod5.gfp:kanMX6 leu1.32 ura4.d18	This Study
tea1::URA4 tea2.gfp:kanMX6 leu1.32 ura4.d18	This Study
tea1::URA4 tea3.gfp:kanMX6 leu1.32 ura4.d18	This Study
tea1::URA4 wsh3.gfp:URA4 leu1.32 ura4.d18	This Study
tea2::hphMX6 for3.gfp:kanMX6 leu1.32 ura4.d18	This Study
tea2::hphMX6 bud6.gfp:kanMX6 leu1.32 ura4.d18	This Study
tea2::hphMX6 bud6.gfp:kanMX6 leu1.32 ura4.d18	This Study
tea2::hphMX6 mal3.gfp:URA4 leu1.32 ura4.d18	This Study
tea2::hphMX6 tip1.4gfp:kanMX6 leu1.32 ura4.d18	This Study
tea2::hphMX6 nmt81mod5.gfp:kanMX6 leu1.32 ura4.d18	This Study
tea2::hphMX6 tea1.gfp:URA4 leu1.32 ura4.d18	This Study
tea2::hphMX6 tea3.gfp:kanMX6 leu1.32 ura4.d18	This Study
tea2::hphMX6 wsh3.gfp:URA4 leu1.32 ura4.d18	This Study
tea3::kanMX6 for3.gfp:kanMX6 leu1.32 ura4.d18	This Study
tea3::kanMX6 bud6.gfp:kanMX6 leu1.32 ura4.d18	This Study
tea3::kanMX6 myo52.gfp:kanMX6 leu1.32 ura4.d18	This Study
tea3::kanMX6 mal3.gfp:kanMX6 leu1.32 ura4.d18	This Study
tea3::kanMX6 tip1.4gfp:kanMX6 leu1.32 ura4.d18	This Study
tea3::kanMX6 mod5.gfp:kanMX6 leu1.32 ura4.d18	This Study
tea3::kanMX6 tea1.gfp:URA4 leu1.32 ura4.d18	This Study
tea3::kanMX6 tea2.gfp:kanMX6 leu1.32 ura4.d18	This Study
tea3::kanMX6 wsh3.gfp:URA4 leu1.32 ura4.d18	This Study
wsh3::natMX6 for3.gfp:kanMX6 leu1.32 ura4.d18	This Study
wsh3::natMX6 bud6.gfp:kanMX6 leu1.32 ura4.d18	This Study
wsh3::natMX6 myo52.gfp:kanMX6 leu1.32 ura4.d18	This Study

wsh3::natMX6 mal3.gfp:URA4 leu1.32 ura4.d18	This Study
wsh3::natMX6 tip1.4gfp:kanMX6 leu1.32 ura4.d18	This Study
wsh3::natMX6 nmt81mod5.gfp:kanMX6 leu1.32 ura4.d18	This Study
wsh3::natMX6 tea1.gfp:URA4 leu1.32 ura4.d18	This Study
wsh3::natMX6 tea2.gfp:kanMX6 leu1.32 ura4.d18	This Study
wsh3::natMX6 tea3.gfp:kanMX6 leu1.32 ura4.d18	This Study
for3.gfp:kanMX6 sid4.tomato:hphMX6 leu1.32 ura4.d18	This Study
bud6.gfp:kanMX6 sid4.tomato:hphMX6 leu1.32 ura4.d18	This Study
myo52.gfp:kanMX6 sid4.tomato:hphMX6 leu1.32 ura4.d18	This Study
mal3.gfp:URA4 sid4.tomato:hphMX6 leu1.32 ura4.d18	This Study
tip1.4gfp:kanMX6 sid4.tomato:hphMX6 leu1.32 ura4.d18	This Study
nmt81mod5.gfp:kanMX6 sid4.tomato:hphMX6 leu1.32 ura4.d18	This Study
tea1.gfp:URA4 sid4.tomato:hphMX6 leu1.32 ura4.d18	This Study
tea2.gfp:kanMX6 sid4.tomato:hphMX6 leu1.32 ura4.d18	This Study
tea3.gfp:kanMX6 sid4.tomato:hphMX6 leu1.32 ura4.d18	This Study
wsh3.gfp:URA4 sid4.tomato:hphMX6 leu1.32 ura4.d18	This Study

References

1 Feierbach B & Chang F (2001) Roles of the fission yeast formin for3p in cell polarity, actin cable formation and symmetric cell division. *Curr. Biol.* **11**, 1656–1665.

2 Feierbach B, Verde F & Chang F (2004) Regulation of a formin complex by the microtubule plus end protein tea1p. *The Journal of Cell Biology* **165**, 697–707.

3 Win TZ, Gachet Y, Mulvihill DP, May KM & Hyams JS (2001) Two type V myosins with non-overlapping functions in the fission yeast Schizosaccharomyces pombe: Myo52 is concerned with growth polarity and cytokinesis, Myo51 is a component of the cytokinetic actin ring. *J. Cell. Sci.* **114**, 69–79.

4 Grallert A, Beuter C, Craven RA, Bagley S, Wilks D, Fleig U & Hagan IM (2006) S. pombe CLASP needs dynein, not EB1 or CLIP170, to induce microtubule instability and slows polymerization rates at cell tips in a dynein-dependent manner. *Genes & Development* **20**, 2421–2436.

5 Coll PM, Trillo Y & Ametzazurra A (2003) Gef1p, a new guanine nucleotide exchange factor for Cdc42p, regulates polarity in Schizosaccharomyces pombe. *Molecular biology of the …* **14**, 313–323.

6 Snaith HA & Sawin KE (2003) Fission yeast mod5p regulates polarized growth through anchoring of tea1p at cell tips. *Nature* **423**, 647–651.

7 Mata J & Nurse P (1997) tea1 and the microtubular cytoskeleton are important for generating global spatial order within the fission yeast cell. *Cell* **89**, 939–949.

8 Arellano M, Niccoli T & Nurse P (2002) Tea3p is a cell end marker activating polarized growth in Schizosaccharomyces pombe. *Current Biology* **12**, 751–756.

9 Alvarez-Tabarés I, Grallert A, Ortiz J-M & Hagan IM (2007) Schizosaccharomyces pombe protein phosphatase 1 in mitosis, endocytosis and a partnership with Wsh3/Tea4 to control polarised growth. *J. Cell. Sci.* **120**, 3589–3601.

10 Browning H, Hayles J, Mata J, Aveline L, Nurse P & McIntosh JR (2000) Tea2p is a kinesin-like protein required to generate polarized growth in fission yeast. *The Journal of Cell Biology* **151**, 15–28.
