# Supplementary material for: Dependency relationships within the fission yeast polarity network
Source: FEBS Lett. 2018 Jul 13;592(15):2543–9. doi: 10.1002/1873-3468.13180 (PMC6120479; doi:10.1002/1873-3468.13180)
Supplement: Supplementary file 2 — Fig. S1. Polarity marker localisation dependency of Bud6‐GFP. Fig. S2. Polarity marker localisation dependency of For3‐GFP. Fig. S3. Polarity marker localisation dependency of Mod5‐GFP. Fig. S4. Polarity marker localisation dependency of Myo52‐GFP. Fig. S5. Polarity marker localisation dependency of Tea1‐GFP. Fig. S6. Polarity marker localisation dependency of Tea2‐GFP. Fig. S7. Polarity marker localisation dependency of Tea3‐GFP. Fig. S8. Polarity marker localisation dependency of Tip1‐3GFP. Fig. S9. Polarity marker localisation dependency of Tea4(Wsh3)‐GFP. [file FEB2-592-2543-s002.pdf]

Bud6-GFP

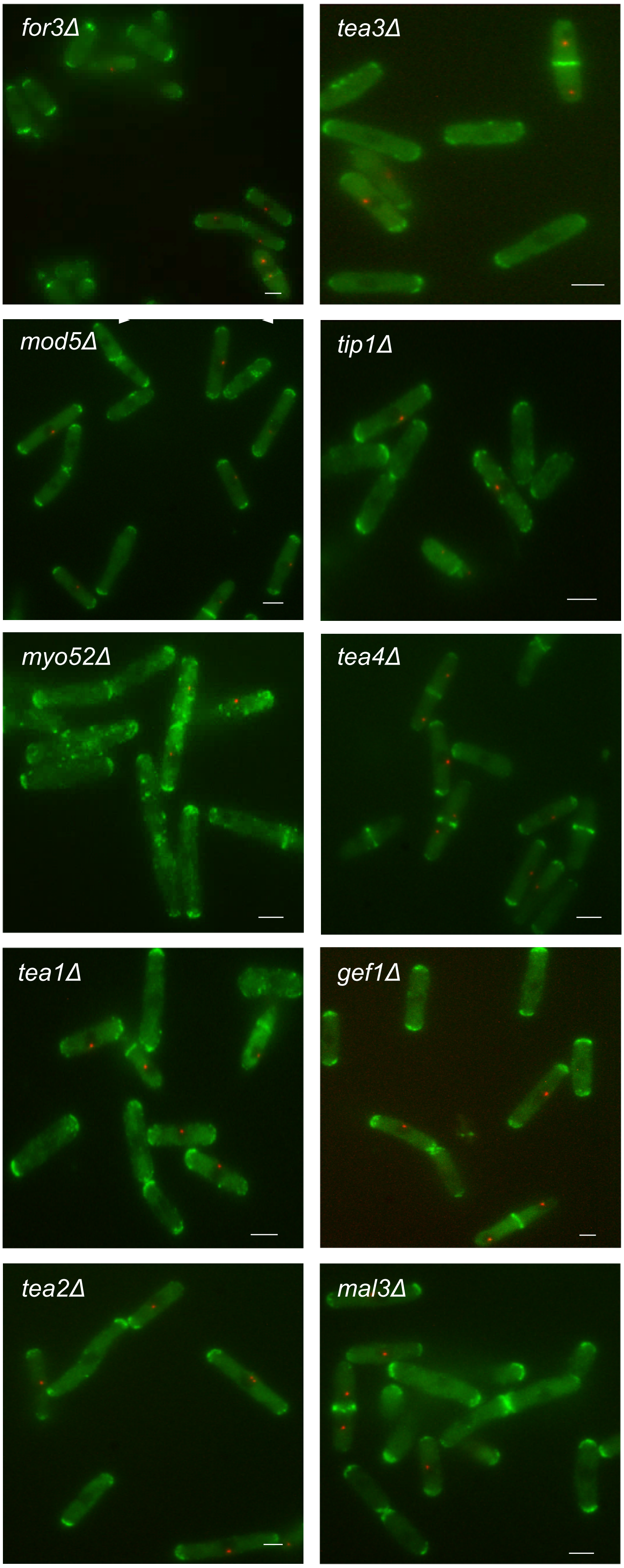

Supplementary Figure 1

For3-GFP

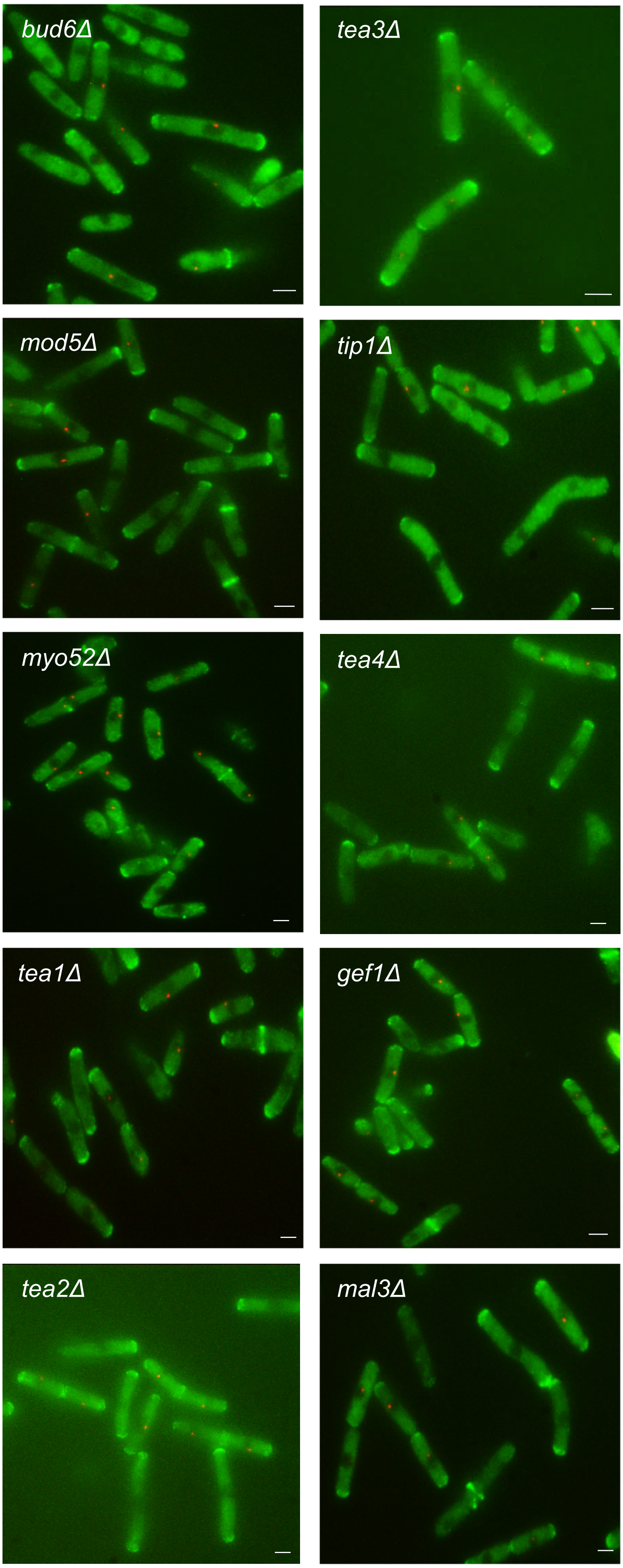

Supplementary Figure 2

Mod5-GFP

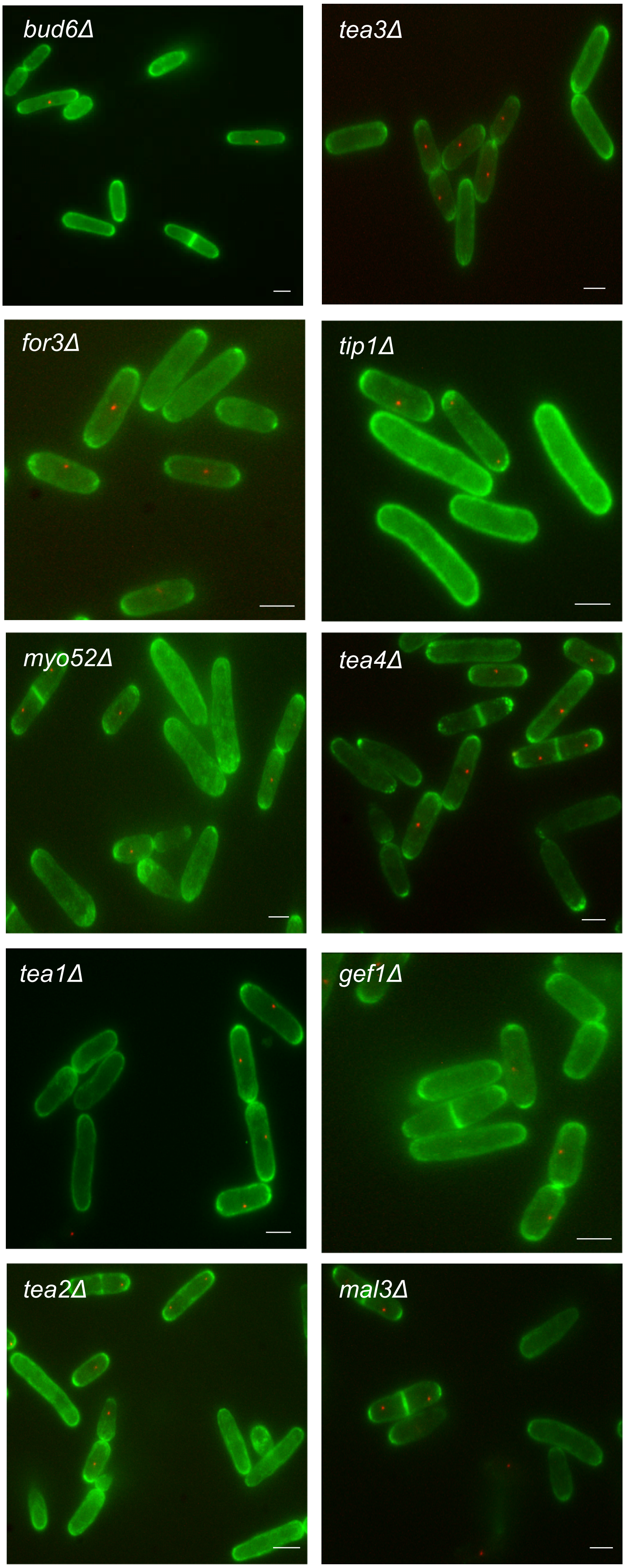

Supplementary Figure 3

Myo52-GFP

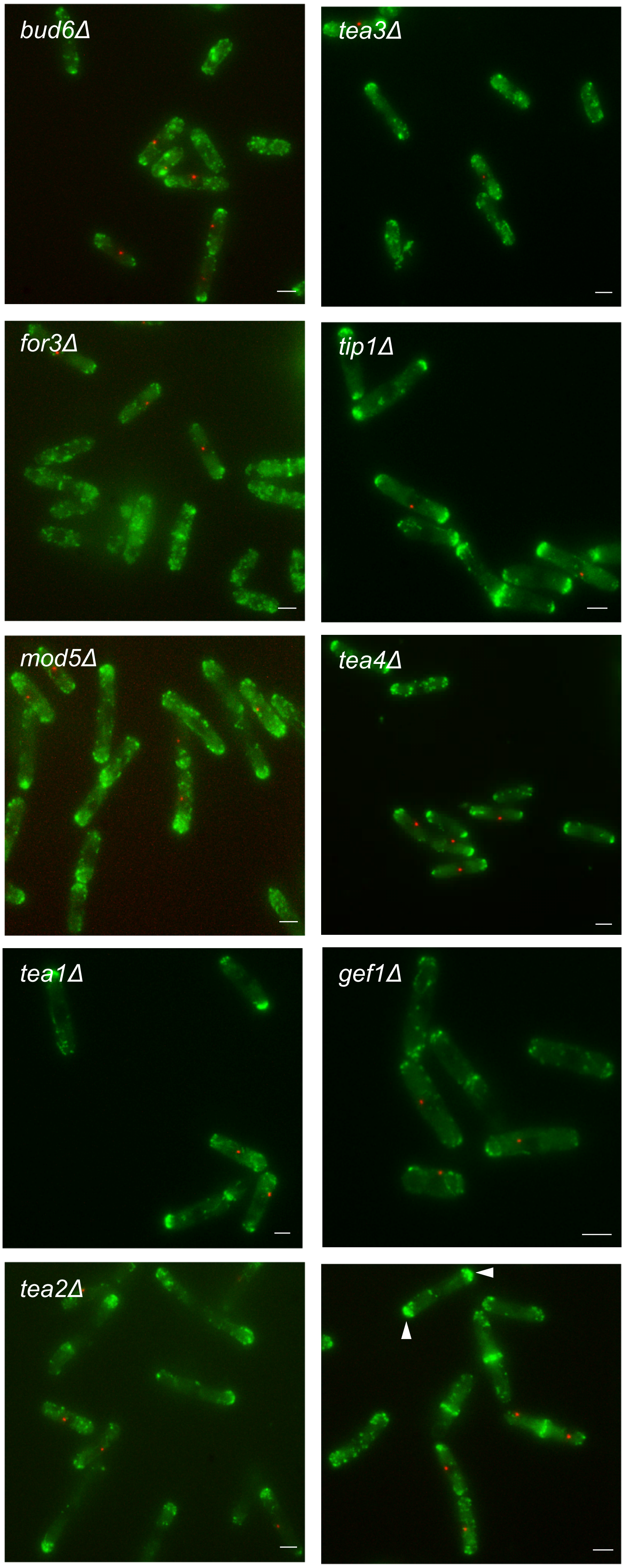

Supplementary Figure 4

Tea1-GFP

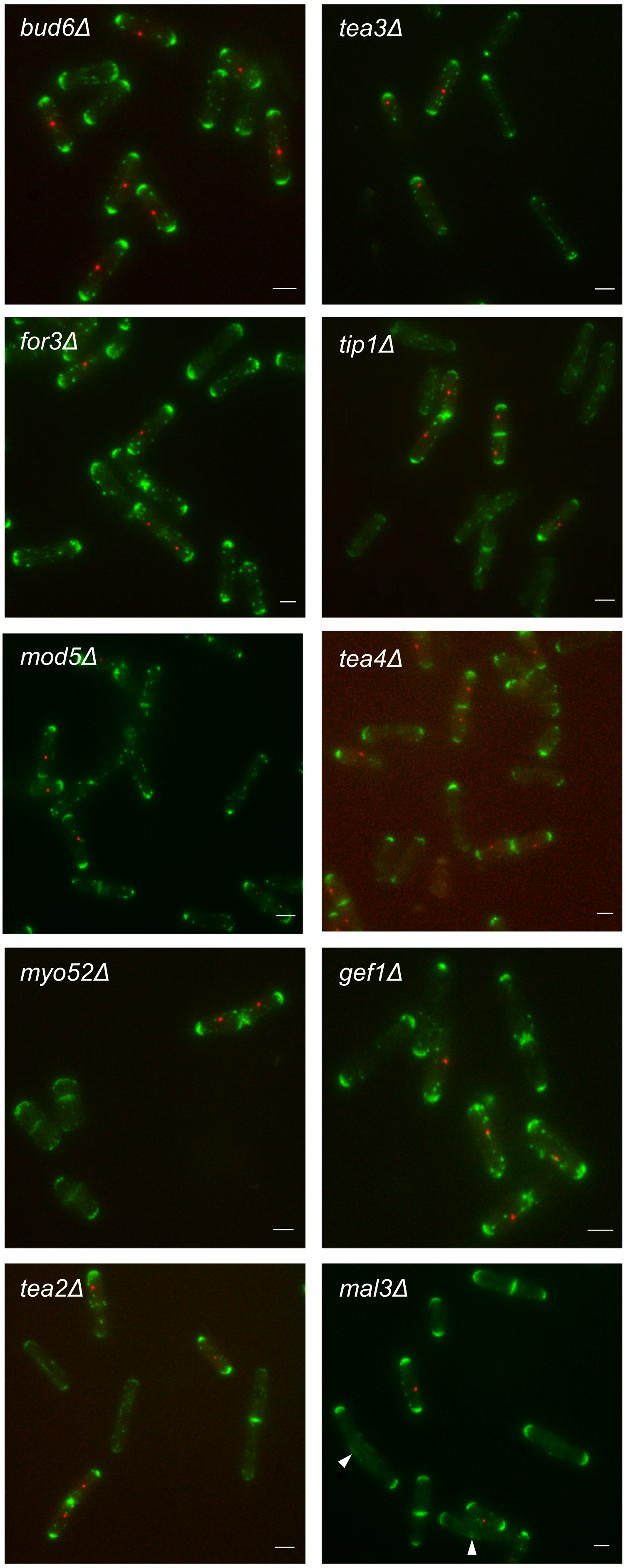

Supplementary Figure 5

Tea2-GFP

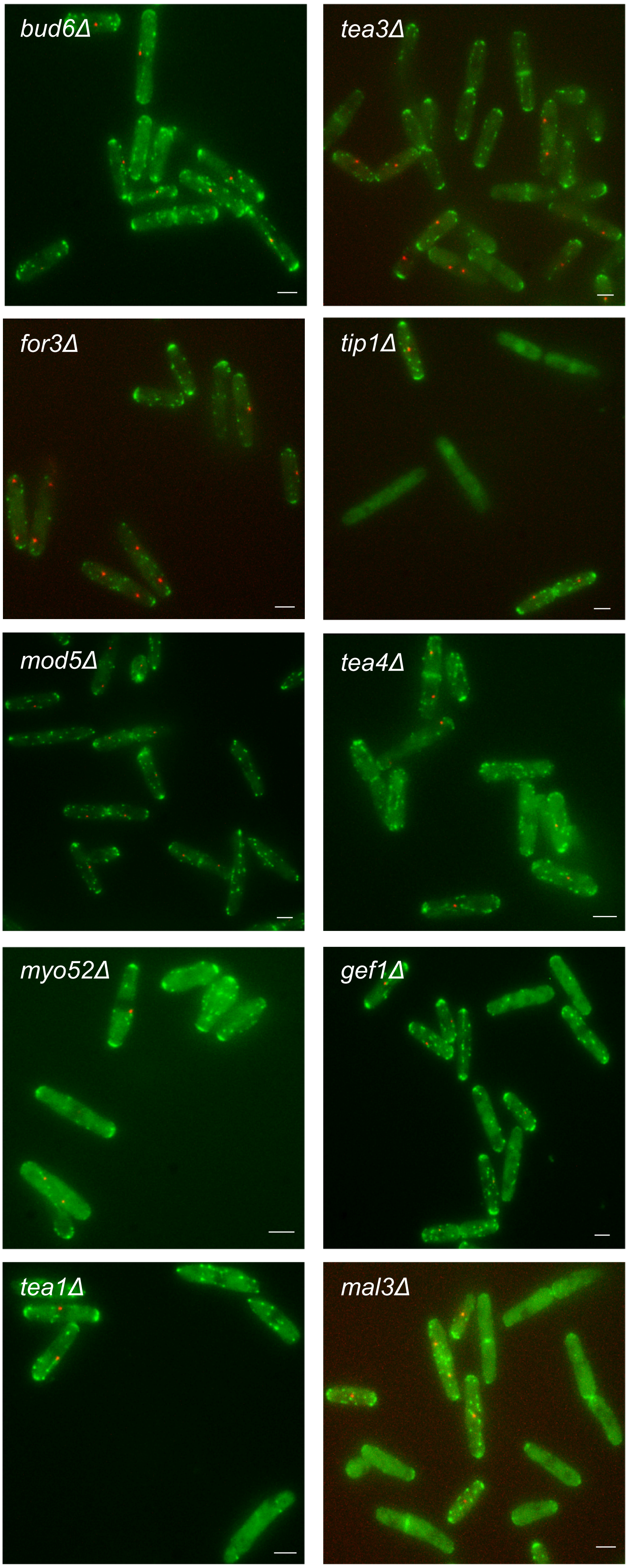

Supplementary Figure 6

Tea3-GFP

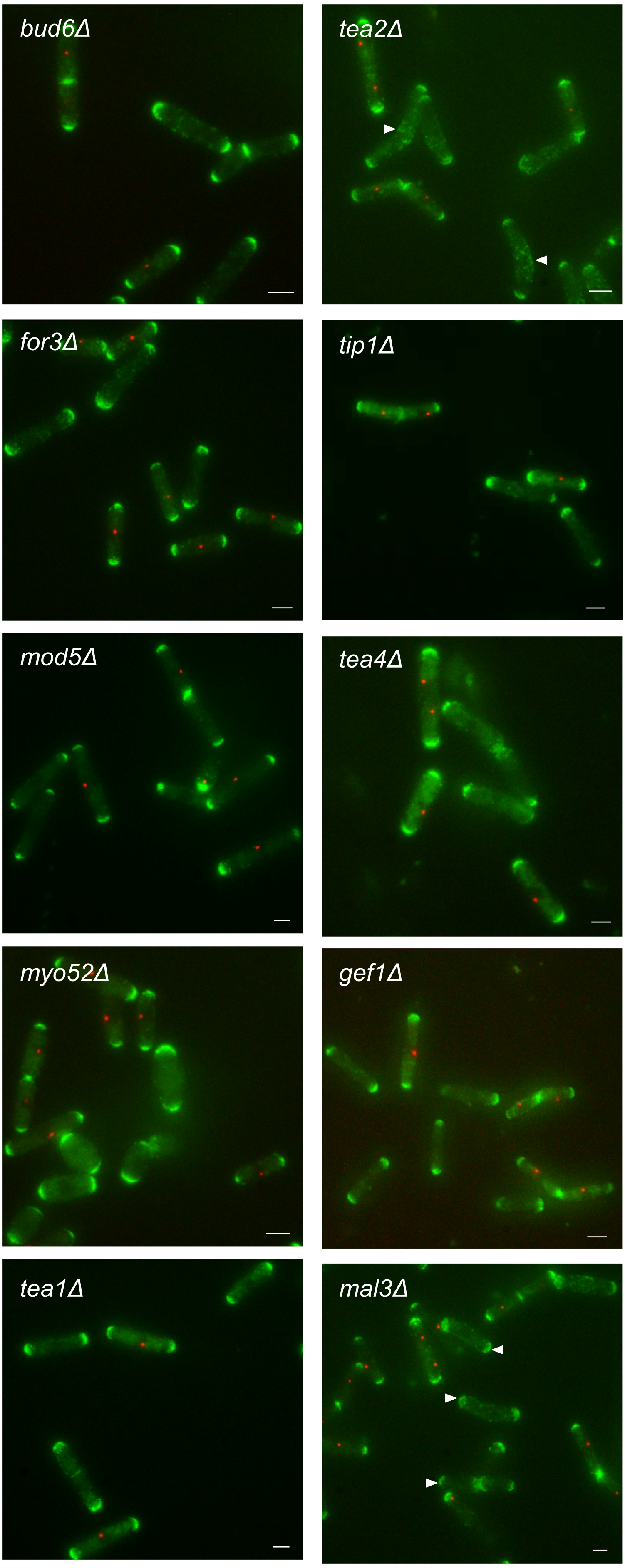

Supplementary Figure 7

Tip1-3GFP

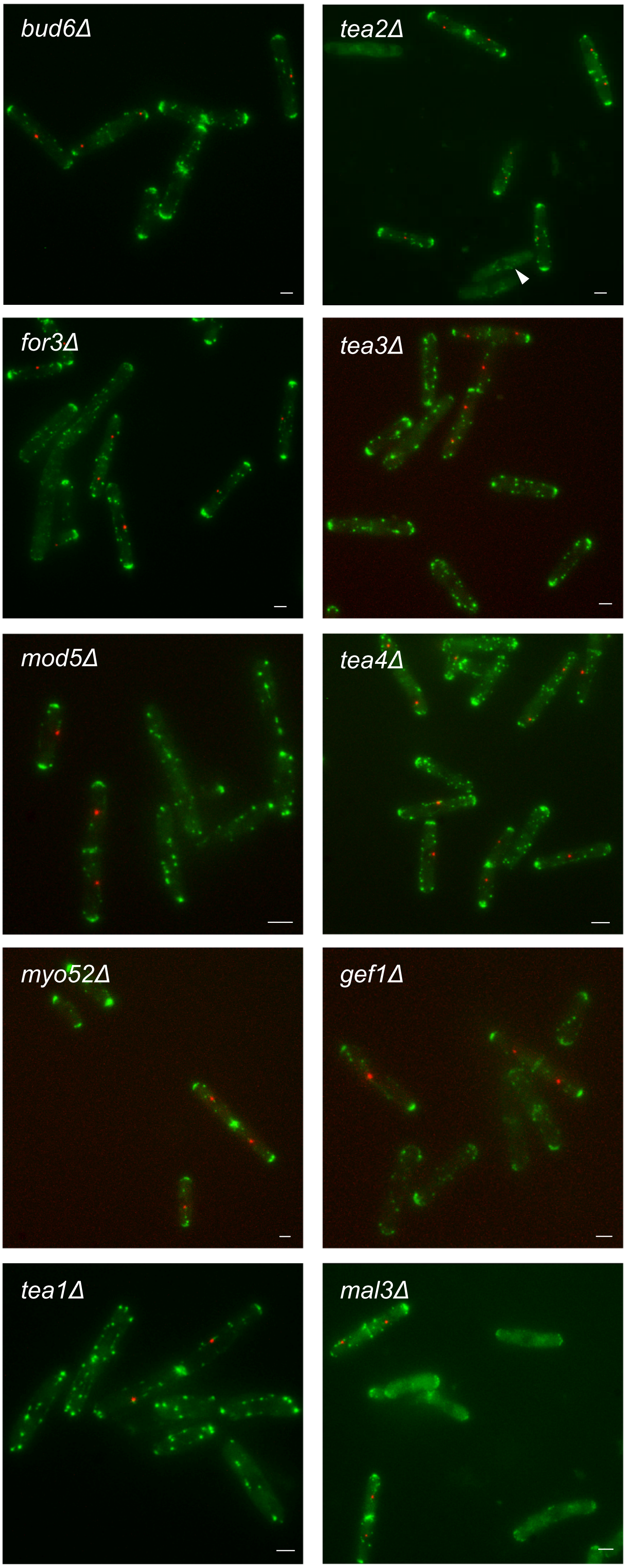

Supplementary Figure 8

Tea4-GFP

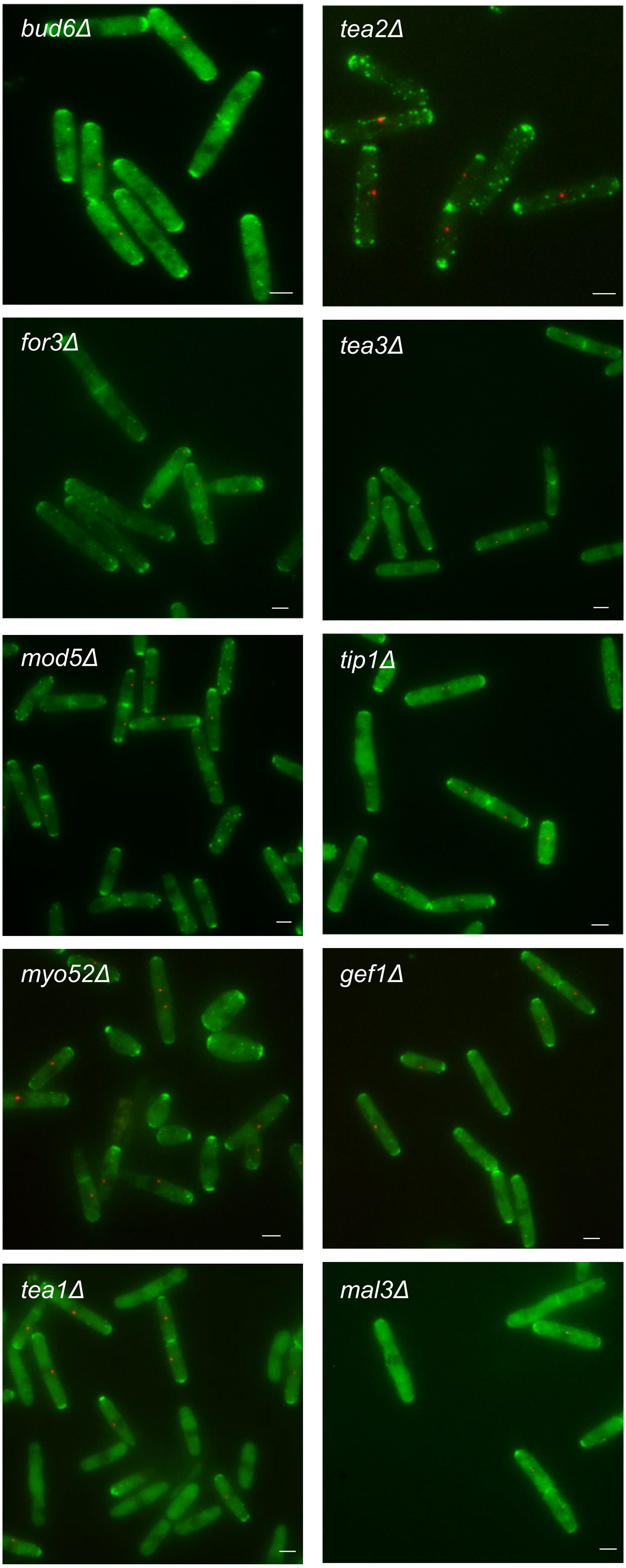

Supplementary Figure 9
