# Supplementary material for: Dependency relationships within the fission yeast polarity network
Source: FEBS Lett. 2018 Jul 13;592(15):2543–9. doi: 10.1002/1873-3468.13180 (PMC6120479; doi:10.1002/1873-3468.13180)
Supplement: Supplementary file 3 [file FEB2-592-2543-s003.pdf]

Supplementary Figure Legends

Figure S1. Polarity marker localisation dependency of Bud6-GFP. Typical maximum projection images of Bud6-GFP (green) signal in wild type cells co-expressing Sid4-tdTomato (red) mounted onto coverslips with each polarity deletion strains (no red signal). Scale: 5μm.

Figure S2. Polarity marker localisation dependency of For3-GFP. Typical maximum projection images of For3-GFP (green) signal in wild type cells co-expressing Sid4-tdTomato (red) mounted onto coverslips with each polarity deletion strains (no red signal). Scale: 5μm.

Figure S3. Polarity marker localisation dependency of Mod5-GFP. Typical maximum projection images of Mod5-GFP (green) signal in wild type cells co-expressing Sid4-tdTomato (red) mounted onto coverslips with each polarity deletion strains (no red signal). Scale: 5μm.

Figure S4. Polarity marker localisation dependency of Myo52-GFP. Typical maximum projection images of Myo52-GFP (green) signal in wild type cells co-expressing Sid4-tdTomato (red) mounted onto coverslips with each polarity deletion strains (no red signal). Scale: 5μm.

Figure S5. Polarity marker localisation dependency of Tea1-GFP. Typical maximum projection images of Tea1-GFP (green) signal in wild type cells co-expressing Sid4-tdTomato (red) mounted onto coverslips with each polarity deletion strains (no red signal). Scale: 5μm.

Figure S6. Polarity marker localisation dependency of Tea2-GFP. Typical maximum projection images of Tea2-GFP (green) signal in wild type cells co-expressing Sid4-tdTomato (red) mounted onto coverslips with each polarity deletion strains (no red signal). Scale: 5μm.

Figure S7. Polarity marker localisation dependency of Tea3-GFP. Typical maximum projection images of Tea3-GFP (green) signal in wild type cells co-expressing Sid4-tdTomato (red) mounted onto coverslips with each polarity deletion strains (no red signal). Scale: 5μm.

Figure S8. Polarity marker localisation dependency of Tip1-3GFP. Typical maximum projection images of Tip1-3GFP (green) signal in wild type cells co-expressing Sid4-tdTomato (red) mounted onto coverslips with each polarity deletion strains (no red signal). Scale: 5μm.

Figure S9. Polarity marker localisation dependency of Tea4(Wsh3)-GFP. Typical maximum projection images of Tea4(Wsh3)-GFP (green) signal in wild type cells co-expressing Sid4-tdTomato (red) mounted onto coverslips with each polarity deletion strains (no red signal). Scale: 5μm.
